# Targeting ferroptosis, the achilles’ heel of breast cancer: A review

**DOI:** 10.3389/fphar.2022.1036140

**Published:** 2022-11-16

**Authors:** Yang Liu, Yueting Hu, Yi Jiang, Jiawen Bu, Xi Gu

**Affiliations:** Department of Oncology, Shengjing Hospital of China Medical University, Shenyang, China

**Keywords:** iron, breast cancer, ferroptosis, therapy, lipid

## Abstract

Ferroptosis is referred as a novel type of cell death discovered in recent years with the feature of the accumulation of iron-dependent lipid reactive oxygen species. Breast cancer is one of the most common malignant cancers in women. There is increasing evidence that ferroptosis can inhibit breast cancer cell growth, improve the sensitivity of chemotherapy and radiotherapy and inhibit distant metastases. Therefore, ferroptosis can be regarded a new target for tumor suppression and may expand the landscape of clinical treatment of breast cancer. This review highlights the ferroptosis mechanism and its potential role in breast cancer treatment to explore new therapeutic strategies of breast cancer.

## 1 Introduction

Ferroptosis is a kind of regulated cell death (RCD) characterized by iron dependent-mechanisms and excessive lipid peroxidation (Stockwell et al., 2017). It participates in tumor progression and disorders of the nervous system and cardiovascular system (Tang et al., 2021). However, despite breakthroughs in cancer treatment, the therapeutic response of cancer is limited by the acquired and intrinsic resistance of the tumor to apoptosis. Therefore, targeting non-apoptotic cell death offers a new option for cancer treatment (Hanahan and Weinberg, 2011). Unique metabolism, high levels of reactive oxygen species (ROS), and related gene mutations confer cancer cells inherently prone to the ferroptosis, while other cancer cells appear to rely particularly on the defense system of ferroptosis to survive under metabolic and oxidative stress conditions (Chen X. et al., 2021). Therefore, the destruction of these defense systems will cause fatal damage to these cancer cells, while normal cells will not be affected. Ferroptosis is also considered an important cell death mechanism caused by a number of therapies, including chemotherapy, radiotherapy (RT), targeted therapy and immunotherapy (Hassannia et al., 2019). It is also found that ferroptosis plays a controversial role in immunogenic cell death (ICD). The tumor cells with ferroptosis could diminish anti-tumor immune response by inhibiting the antigen presenting cells (Sacco et al., 2021; Wiernicki et al., 2022). However, the ferroptosis inducers (FINS) could enhance the efficacy of conventional treatment has been regarded as the potential drugs in cancer treatment (Mou et al., 2019). Breast cancer is currently the tumor with the highest incidence rate in the world (Duggan et al., 2021). The breast is in a unique microenvironment with plentiful adipocytes infiltrating. Previous studies have shown that adipocytes can regulate fatty acid metabolism, enhance the invasion and metastasis of breast cancer. Ferroptosis has been shown to participate in the development and treatment of breast cancer. Studies have found that highly invasive triple negative breast cancer (TNBC) cells are susceptive to ferroptosis and are particularly vulnerable to FINs. The susceptibility is attributed to several metabolic characteristics including high polyunsaturated fatty acids (PUFA) levels, expanded liable iron pool, and weakened Glutathione Peroxidase 4 (GPX4)-glutathione (GSH) defense system. The ferroptosis induced by the drugs in breast cancer is a potential and valuable research direction, which indicates that the it is an attractive target for the treatment of “refractory” tumors (Sui et al., 2022). We summarize the mechanisms of ferroptosis and explore its prospective on breast cancer in the review, which hopefully will provide novel strategies to improve the therapeutic response of breast cancer.

## 2 Mechanism of ferroptosis in breast cancer

**FIGURE 1:**
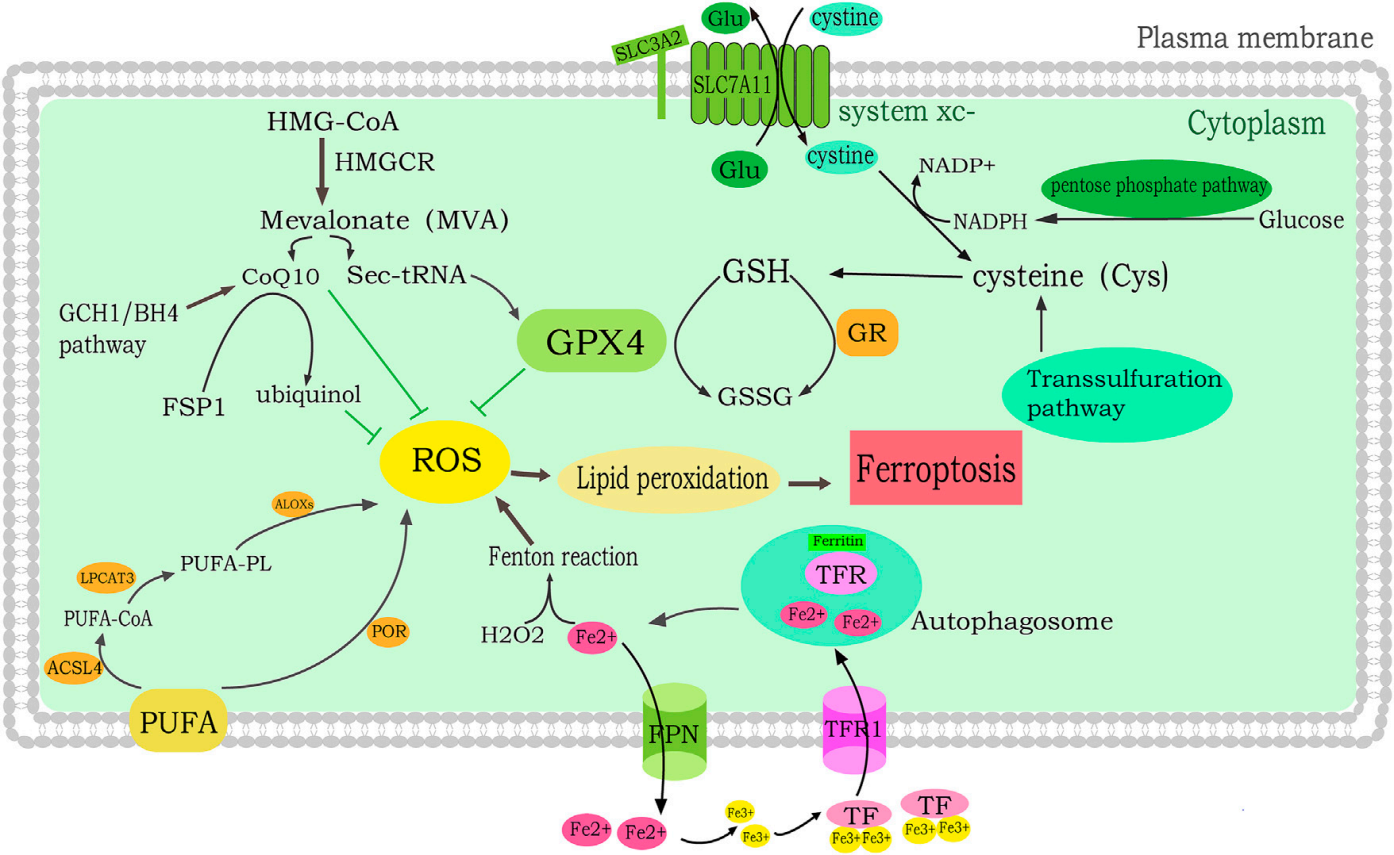
Molecular mechanisms of ferroptosis in breast cancer. lipid peroxidation leads to ferroptosis. PUFA is catalyzed by LPCAT3, ACSL4 and LOX to generate PUFA-PLOOH, which induces ferroptosis. In addition, PUFAs also promote ferroptosis through POR-mediated conversion to lipid peroxides. The MVA pathway suppresses ROS by regulating Sec-tRNA and CoQ10. The iron mediates ferroptosis. FPN regulates the intracellular iron level by converting Fe^2+^ to extracellular iron. TF binds to the TFR to convert Fe^3+^ to Fe^2+^ and enter cells. Free Fe^2+^ then induces the lipid ROS production by Fenton reaction.

Ferroptosis is characterized by serious damage to mitochondrial morphology including volume shrinkage, condensed bilayer membrane density and ballooning phenotype (a clear, rounded cell consisting mainly of empty cytosol) (Battaglia et al., 2022). Ferroptosis is the result of antagonism of its prerequisites (drivers) and defense systems (Dixon et al., 2012). Prerequisites for ferroptosis include iron metabolism, mitochondrial metabolism, synthesis of polyunsaturated fatty acid phospholipid (PUFA-PL) and lipid peroxidation (Tang and Kroemer, 2020). The ferroptosis defense system mainly includes the GPX4/GSH system, FSP1-CoQH2 system, DHODH-CoQH2 system and GCH1-BH4 system. Ferroptosis occurs when the drivers of ferroptosis significantly exceed the detoxification capabilities of the defense system. When the dysregulation of iron hemostasis induce free iron increasing in cells, ROS is catalyzed by free iron through Fenton reaction, thus promotes lipid peroxidation, accumulates lipid peroxides and ultimately induces ferroptosis. System Xc–, the cystine/glutamate reverse transporter, participates in the GSH synthesis. GPX4 utilizes GSH as substrate to reduce lipid peroxides, decrease the accumulation of lipid peroxides, and inhibit the occurrence of ferroptosis (Lei et al., 2022); (Figure 1).

### 2.1 Drivers of ferroptosis in breast cancer

#### 2.1.1 Iron metabolism

Increased intracellular iron induces ROS production through the Fenton reaction, implying that iron overload may be an activator of ferroptosis. Ferroptosis sensitivity can be improved by increased iron uptake, reduced iron consumption and impaired cellular iron export (Liu et al., 2020). The iron is mainly stored in ferritin in the form of bound iron. Ferritinophagy releases the iron from ferritin into the liable iron pool which induce the ferroptosis. Tranferrin (TF) binds to the Tranferrin receptor to convert Fe^3+^ to Fe^2+^. Ferroportin (FPN) is required for transferring excess Fe^2+^ out of cells. FPN knocking out can induced ferroptosis by iron overload. FPN is lower in breast cancer patients compared with healthy people, which has great impact on the progression of breast cancer (Pinnix et al., 2010).

#### 2.1.2 Mitochondrial reactive oxygen species

FINs can promote the production of mitochondrial ROS (MitoROS) to make mitochondria disappearance and ATP depletion by the unfolding of mitochondrial permeability transition pore (MPTP). MitoROS plays a positive feedback role in the process of mitochondrial homeostasis and ferroptosis. The impairment of CDGSH Iron Sulfur Domain 2 (CISD2) function in breast cancer can induce the destruction of mitochondrial labile iron pool, which improve the mitochondrial ROS level, thus leading to the activation of ferroptosis (Karmi et al., 2021).

#### 2.1.3 PUFAs and lipid peroxidation

PUFAs can be oxidized by ROS to produce unstable lipid hydroperoxide (LOH/LOOH) and lipid peroxide. Studies have shown that with the increased intake of PUFAs in diet, the level of ferroptosis-related genes such as GPX4, SLC7A11, DHFR and FSP1 in tumor cells has changed, which can promote the lipid peroxidation of cancer cells in the acidic tumor microenvironment, thus induce the ferroptosis in cancer cells (Dierge et al., 2021). ACSL4 and LPCAT3 are two key enzymes in the formation of lipid peroxides. Some studies have found that the level of lipid oxidation in breast cancer plasma and tumor tissue is negatively related to the survival. The expression of ACSL4 in TNBC is related to the sensitivity to ferroptosis. Studies have shown that ACSL4 is underlying enriched in basal-like subtypes and regulate the enrichment of long polyunsaturated–ω-6 fatty acids on cell membrane to inpede the ferroptosis (Doll et al., 2017). Some lipoxygenases (LOXs) are non heme dioxygenases which can oxidize the PUFAs to trigger the ferroptosis. The serum ALOX5 level in breast cancer patients was two times higher than that in the healthy people. It was also found that ALOX5 is positively correlated with tumor stage of breast cancer which could accelerate the growth of breast cancer cells (Kumar et al., 2016). Recent studies suggest that cytochrome P450 oxidoreductase (POR) also facilitates the lipid peroxidation (Zou et al., 2020).

### 2.2 Defense system of ferroptosis in breast cancer

Lipid oxidation is strictly regulated by the antioxidant defense system. GPX4 is considered to be a vital antioxidant enzyme, which directly eliminates hydroperoxides in lipid bilayers, thus preventing lethal lipid ROS production (Yang et al., 2014). GSH/GPX4 pathway, FSP1/COQ10/NADPH pathway, DHODH pathway and GCH1/BH4 pathway are identified as defense system in ferroptosis.

#### 2.2.1 GSH/GPX4 pathway

GSH/GPX4 pathway includes cystine (Cys) import through System Xc-, the production of cysteine *via* the transsulfuration pathway, and production of selenocysteine through the mevalonate pathway (Ingold et al., 2018). ROS neutralization is catalyzed by GSH during the occurrence of ferroptosis. Selective anti-GSH biosynthesis can be considered to target the metabolic vulnerability ER + breast cancer (Xiong et al., 2019). GSH is found in higher level in tumor lesion than in noncancerous tissue and has been shown to increase the risk of breast cancer (Mehdi et al., 2018). When system Xc- is prohibited, it prevents cystine from entering the cells and blocking GSH synthesis, leading to the inactivation of GPX4, resulting in the accumulation of Lip-ROS (Lee et al., 2021). The subtypes of TNBC have different characteristics of ferroptosis. Luminal androgen receptor (LAR) subtype is the most vulnerable to the ferroptosis treatment. The GPX4 which is driven by androgen receptor is a key factor of ferroptosis in LAR subtype breast cancer. The GPX4 inhibitor not only suppress the proliferation but also reshape the tumor microenvironment of LAR subtype (Yang F. et al., 2022).

#### 2.2.2 Ferroptosis suppressor protein 1/coenzyme Q 10/NAD(P)H pathway

Ferroptosis suppressor protein 1 (FSP1), a suppressor of ferroptosis, acts as a NADH-dependent coenzyme Q oxidoreductase (Doll et al., 2019). Coenzyme Q 10 (CoQ10) is generated in the mitochondrial electron transport chain by the mevalonate (MVA) pathway, which restrain the mitochondria. 3-hydroxy-methylglutaryl-CoA reductase (HMGCR) is the enzyme that catalyzes HMG-CoA to mevalonate, which increases GPX4 biosynthesis and CoQ10 (Shimada et al., 2016). The inhibition of ferroptosis by FSP1 is mediated by CoQ10. The reduced form of CoQ10 captures lipid peroxidative radicals, while FSP1 catalyzes CoQ10 by using NAD(P)H. The FSP1-CoQ10-NAD(P)H pathway is independent and cooperates with the GSH/GPX4 pathway to defense ferroptosis (Bersuker et al., 2019). Statins can inhibit HMGCR and reduce FSP1-mediated ferroptosis resistance which could be considered as a potential drug for the treatment of TNBC (Yao et al., 2021).

#### 2.2.3 Dihydrolactate dehydrogenase signaling pathway

Dihydrolactate dehydrogenase (DHODH) is located in the inner membrane of mitochondria which is a flavin-dependent enzyme. The DHODH is mainly to catalyze pyrimidine nucleotide synthesis pathway. The electrons are transferred in the mitochondria membrane to make ubiquinone convert to dihydroubiquinone, which induces resistance to ferroptosis in mitochondria. It is suggested that DHODH may regulate ferroptosis independently of the pyrimidine nucleotide synthesis function (Mao et al., 2021). Moreover, the DHODH inhibition could reduce the production of ATP and endogenous ROS and promote the cell cycle arrest in S phase of breast cancer cells (Mohamad Fairus et al., 2017).

### 2.3 Guanosine triphosphate cyclohydrolase 1/BH4 signaling pathway

Guanosine triphosphate cyclohydrolase 1 (GCH1) is a rate-limiting enzyme for tetrahydrobiopterin BH4 synthesis to inhibit ferroptosis independently of GPX4. BH4 is an effective free radical trapping antioxidant that can prevent lipid peroxidation and can be regenerated by dihydrofolate reductase (DHFR). The inhibition of DHFR cooperates with GPX4 deletion could induce ferroptosis (Kraft et al., 2020). GCH1 promotes the immunosuppression of TNBC by regulating tryptophan metabolism (Wei et al., 2021). Further studies are needed to clarify the molecular mechanism of GCH1/BH4 in ferroptosis defense of breast cancer.

## 3 Iron and iron-regulatory proteins involved in breast cancer

Sufficient intracellular iron is a necessary condition for ferroptosis (Pinnix et al., 2010). A recent study showed that compared with non TNBC tumor, iron-regulatory genes are significantly overexpressed in TNBC (Table 1). In particular, a large number of low-level iron export transporters are observed in TNBC, accompanied by high-level expression of iron import transferrin receptors. A European randomized controlled study showed there was a positive association between serum transferrin level and the incidence of ER- breast cancer (Hou et al., 2021). A Canadian cohort study showed that dietary iron supplementation was associated with a low risk of breast cancer, and heme iron was positively related with the risk of the ER and or PR positive breast cancer subtypes in postmenopausal women (Chang et al., 2020). High ferroportin and hepcidin expression play a protective role in patients with breast cancer (Pinnix et al., 2010). Transferrin receptor 1 (TFR1), an independent prognostic factor, is positively related to the amounts of immune cells (CD8+ T cells, CD4+ T cells, neutrophils, B cells, macrophages, and dendritic cells) in breast cancer patients (Chen F. et al., 2021).

**TABLE 1:** The iron and iron-regulatory proteins related with the prognosis of breast cancer.

Protein	Sample	Subtype	Role in survival	Expression	Ref
Transferrin	serum	ER-	poor	upregulated	Hou et al. (2021)
Dietery iron		ER-/PR- in Postmenopausal women	poor	upregulated	Chang et al. (2020)
Ferroportin	cell line Tissue	BC	favorable	downregulated	Pinnix et al. (2010)
Hepcidin	cell line Tissue	BC	poor	upregulated	Pinnix et al. (2010)
TFR1	tissue	BC	poor	upregulated	Chen et al. (2021a)
HMOX1,	tissue	BC	poor	upregulated	Zhu et al. (2021)
PEBP1,	Tissue	BC	favorable	downregulated	Zhu et al. (2021)
KEAP1,	Tissue	BC	favorable	downregulated	Zhu et al. (2021)
LPCAT3	Tissue	BC	favorable	downregulated	Zhu et al. (2021)
AKR1C1	tissue	BC	favorable	downregulated	Zhang et al. (2022)

Ferroptosis-related genes (FRGs) are found to be promising targets for the treatment of breast cancer. Two subgroups, a high-risk group and a low-risk group, can be stratified by nine FRGs, namely S*QLE, G6PD, ALDH3A2, SLC1A4, CHAC1, SIAH2, FLT3, EGLN2,* and *SFXN5,* which are associated with histological type, grade, stage, Her-2 status, and subtype of breast cancer. A high-risk subgroup of patients with positive or negative CTLA4 and PD-1 status demonstrated a significant benefit of combined anti-CTLA4 and anti-PD-1 therapy (Li P. et al., 2021). This new genome panel consisting of nine FRGs which are identified to predict the prognosis and immune status of breast cancer patients. The high expression of ferroptosis-related gene aldo-keto reductase family 1member C1 (AKR1C1) was associated with better survival and various immune infiltrating cells amount in breast cancer (Zhang et al., 2021d). Changes in the genes expression involved in the regulation of iron metabolism may help increase the liable iron pool of cells, promote iron-dependent lipid peroxidation, and make TNBC an iron-enriched tumor prone to ferroptosis. Iron overload leads to the reactivation of oxygen components, lipid peroxidation, and DNA damage. Recently, it has been shown that high iron level of the inflammatory microenvironment may promote the progression and metastasis of breast cancer. Heme oxygenase (HO) level was found to have great impact for the onset of ferroptosis in TNBC (Consoli et al., 2022). MYC increases ADHFE1 and induces iron overload in breast cancer, resulting in poor survival (Mishra et al., 2018). Ferroptosis has been shown to be involved in breast cancer brain metastases, *HOMX1, LPCAT3, PEBP1,* and *KEAP1,* and are associated with relapse and overall survival. The ferroptosis score is associated with iron metabolism, interstitial and immune cell numbers, and iron mortality indicators, which can be used as prognostic markers (Zhu et al., 2021). Taken together, these findings indicates that multiple iron-regulatory genes are related with the survival of breast cancer.

## 4 The molecules participate in the regulation of ferroptosis in breast cancer

**FIGURE 2:**
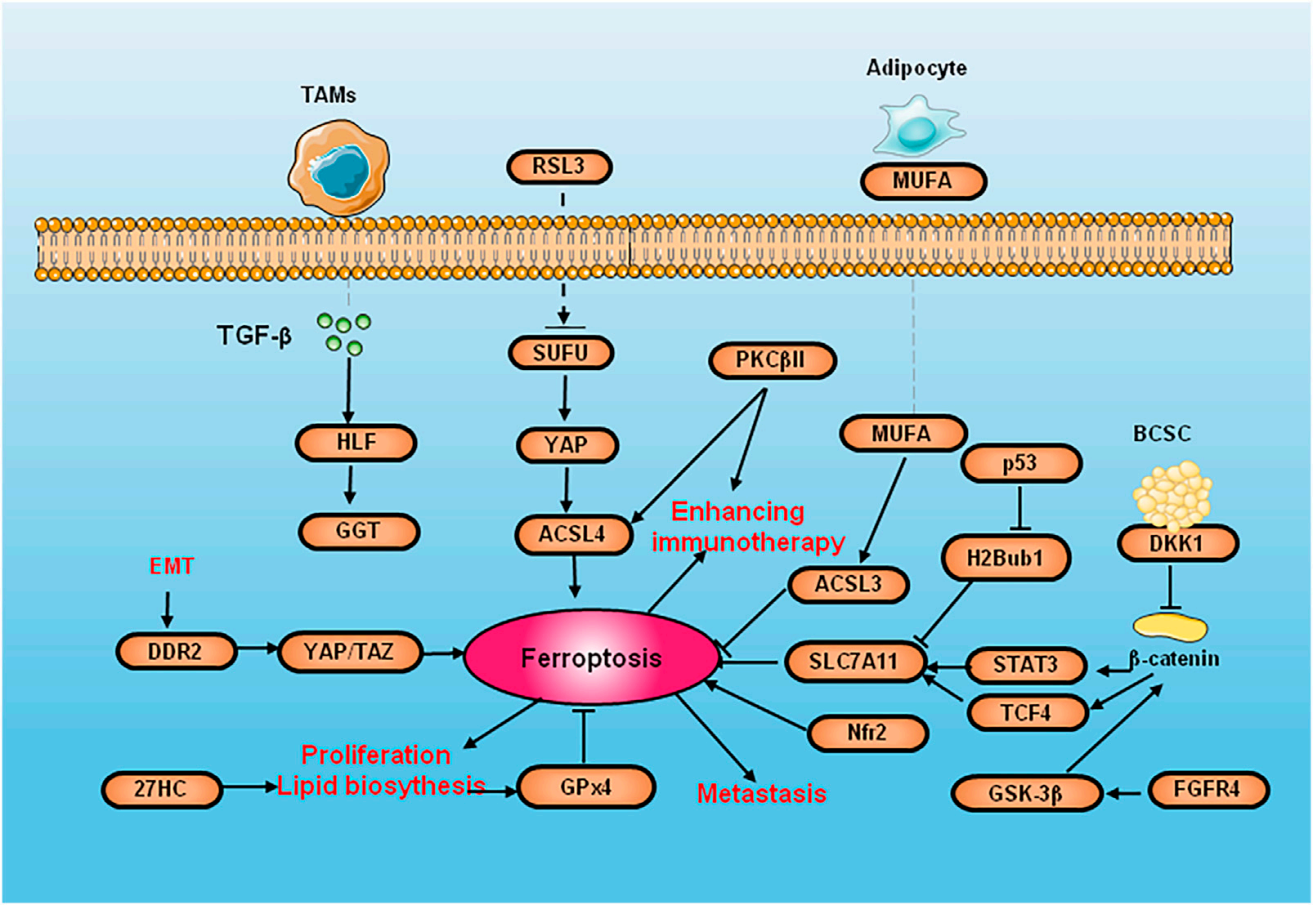
The diagram of cell signaling pathways and molecules of ferroptosis in breast cancer. SUFU deletion increase the ferroptosis by activated by RSL3 *via* ACSL4. PKCβII activate ACSL4 to induce lipid peroxidation and improves the efficacy of immunotherapy. DKK1 inhibit their stem cell properties. Because CSCs are highly sensitive to ferroptosis, DKK1 induces SLC7A11 increasing and promote the development of metastase og CSC. DMOCPTL inhibit GPX4 to induce ferroptosis by EGR-1. 27HC exposure induce the overexpression of GPX4 to inhibit ferroptosis and increase the proliferation and metastasis. The upregulation of DDR2 elicits ferroptosis to recurrent breast cancer through the Hippo pathway. EMT induce DDR2 upregulation to active ferroptodis by regulating YAP/TAZ. GSK-3 β promoting erastin-induced ferroptosis by blocking Nrf2. p53 reduces the expression of SLC7A11 by inhibiting H2Bub1. FGFR4 suppress GSH synthesis and efflux of Fe^2+^ through β-catenin/TCF4/SLC7A1 signaling pathway.

Several cell signaling pathways and molecules can directly or indirectly act on key factors that initiate or regulate ferroptosis, thus positively or negatively regulating ferroptosis (Figure 2).

### 4.1 Driving system of ferroptosis

Nuclear factor erythroid2-related factor 2 (Nrf2) can regulate ferroptosis through p62-Keap1-NRF2 pathway. The activation of p62-Keap1-NRF2 signal pathway can also promote the expression of System Xc-, accelerate the transportation of cystine/glutamic acid, and thus eliminate the accumulated lipid peroxidation. The activation of Nrf2 promotes the iron storage, reduces the iron consumption, increasing the expression of target genes NADH Dehydrogenase Quinone 1 (NQO1) and HO-1 which are related to iron and ROS (Xu et al., 2019). Glycogen synthase kinase-3 β (GSK-3 β) promoting erastin-induced ferroptosis by blocking Nrf2 in breast cancer (Wu et al., 2020). Suppressor of fused homolog (SUFU) is a key element of the Hedgehog pathway (Fang et al., 2022). The deletion of SUFU can increase the ferroptosis of breast cancer cells activated by RSL3 by decreasing ACSL4. Vincristine can promote the RSL3 sensitivity of breast cancer cells by downregulating SUFU. Protein kinase C-βII (PKCβII) is activated by lipid peroxidation during ferroptosis, which can directly phosphorylate the Thr328 site of ACSL4 to activate ACSL4, strengthen the biosynthesis of PUFA-containing lipids, and induce lipid peroxidation. PKCβII induces ferroptosis and improves the response of immunotherapy (Zhang et al., 2022).

### 4.2 Defense system of ferroptosis

Recent studies have shown that cancer stem cells (CSC) are relatively enriched in lung metastases due to their high invasive properties, and enriched CSCs can secrete negative Dickkopf1 (DKK1) feedback to inhibit their stem cell properties. Because CSCs are highly sensitive to ferroptosis, DKK1 induces an increase in SLC7A11 expression and regulates inhibition of CSC properties and thus can protect lung metastatic cells from ferroptosis and promote the development of metastases. In multiple models of breast cancer metastasis, small-molecule inhibitors of DKK1 can almost completely block the occurrence of lung metastases (Wu M. et al., 2022). p53 induces ferroptosis by SLC7A11 (Magri et al., 2021). In fact, mutant p53 inhibits Nrf2-dependent SLC7A11 transcription to promote SLC7A11 expression (Clemons et al., 2017). In addition, p53 promotes the nuclear translocation of ubiquitin-specific protease (USP7) and reduces the expression of SLC7A11 by inhibiting H2B monoubiquitination (H2Bub1) (Wang Y. et al., 2019).

DMOCPTL can induce EGR1-mediated apoptosis and ferroptosis and in TNBC by GPX4 ubiquitination. Long-term exposure to the cholesterol metabolite 27-hydroxycholesterol (27HC) causes breast cancer cells to be resistant to 27HC and overexpress GPX4, which inhibits ferroptosis and increases tumorigenicity and tumor metastasis (Liu W. et al., 2021). Up-regulation of the discoidin domain receptor 2 (DDR2) elicits the susceptibility of ferroptosis by GSH consumption of recurrent breast cancer through the Hippo pathway. Epithelial mesenchymal transition-driven upregulation of DDR2 suppresses tumor proliferation in recurrent breast cancer by regulating YAP/TAZ-mediated ferroptosis (Lin et al., 2021). The inhibiting of Fibroblast Growth Factor Receptor 4 (FGFR4) mediated by m^6^A hypomethylation significantly suppress GSH synthesis and efflux of Fe^2+^ through β-catenin/TCF4-SLC7A11/FPN1 signaling pathway, which trigger the excessive production of ROS and abnormal iron pool accumulation in Her-2 positive breast cancer (Zou et al., 2022).

### 4.3 The regulation of tumor microenvironment in ferroptosis of breast cancer

The tumor microenvironment (TME), especially immune cells, has an important impact on ferroptosis in cancer cells. Interferon-γ (IFN-γ) secreted by CD8+ cytotoxic T cells inhibits cystine uptake by cancer cells by downregulating SLC7A11, thus triggering lipid peroxidation and ferroptosis in various tumors (Wang W. et al., 2019). Tumor-associated macrophages (TAM) secrete transforming growth factor beta 2 (TGF-β2) to regulate liver leukemia factor (HLF). HLF inhibits ferroptosis by activating GGT1 and induces TNBC chemoresistance (Li H. et al., 2022). Adipocytes induce ferroptosis resistance in breast cancer by secreting specific fatty acids. Mono-unsaturated Fatty Acid (MUFA) regulates breast cancer cells by binding with acyl-CoA synthetase long chain family member 3 (ACSL3) to phospholipids, and it is confirmed that the regulation of ferroptosis in breast cancer by adipocytes and exogenous MUFA is dependent on ACSL3 (Xie et al., 2022).

### 4.4 Non-coding RNAs in ferroptosis of breast cancer

**FIGURE 3:**
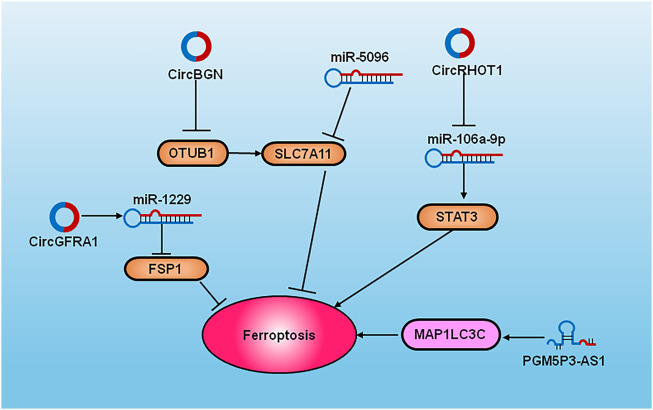
The diagram of cell signaling pathways of ncRNAs and ferroptosis in breast cancer. The overexpression of SLC7A11 inhibits miR-5096 to induce ferroptosis. PGM5P3-AS1 regulates MAP1LC3C to induce the occurrence of ferroptosis. CircRHOT1 inhibits ferroptosis by targeting the miR-106a-5p *via* STAT3. CircGFRA1 sponge with miR-1228 and improves FSP1 expression. CircBGN binds to OTUB1 and SLC7A1 and inhibits ferroptosis.

Non-coding RNAs (ncRNAs) are regarded as novel regulators of various cellular processes in cancer (Zuo et al., 2022); (Figure 3). Some recent studies have shown that they participate in the molecular regulation of ferroptosis. The ectopic overexpression of SLC7A11 inhibits miR-5096-mediated ferroptosis and diminishes antitumor effects in breast cancer (Yadav et al., 2021). PGM5P3-AS1 regulates MAP1LC3C to confer ferroptosis and thus inhibits the progression of TNBC (Qi et al., 2022). Circular RNA RHOT1 prohibits the occurrence of ferroptosis by targeting the miR-106a-5p/STAT3 signaling pathway and increases breast cancer proliferation (Zhang H. et al., 2021). CircGFRA1 facilitates progression of the malignant phenotype of Her-2 positive breast cancer by sponging with miR-1228 and improving FSP1 expression (Bazhabayi et al., 2021). Circ-BGN binds to OTUB1 and SLC7A11 and inhibits ferroptosis to confer resistance to trastuzumab in HER-2+ breast cancer (Wang S. et al., 2022). A integrity understanding of these regulatory network might facilicate the discovery of vulnerable therapeutic target of ferroptosis in breast cancer.

## 5 Potential drugs to treat breast cancer through ferroptosis

The development of cancer treatment strategies based on ferroptosis targets is actively in progress. Loading FINS through nanocarriers can effectively reduce the toxicity of drug delivery systems, overcome the drug resistance of cancer cells, and significantly improve the ferroptosis efficacy.

### 5.1 Targeting iron overload

The demand of tumor cells for iron has increased significantly indicating that iron chelators may be an effective anti-cancer strategy iron chelators exert their anticancer potential by consuming intracellular iron, inhibiting DNA synthesis, inducing apoptosis, and oxidative stress. Furthermore, the reduction of intracellular iron through iron chelation can sensitize breast cancer cells to chemotherapy (Tury et al., 2018). For example, deferoxamine (DFO) and deferoxazole (DFX) show potential antitumor activity in breast cancer by limiting iron bioavailability. DFO promote iron uptake to activate TRF1 and divalent metal transporter 1 (DMT1) *via* the IL6/PI3K/AKT axis (Chen et al., 2019). The physiologically achievable concentration of DFO monotherapy significantly active the apoptosis of breast cancer cells and the xenotransplantation of mouse breast tumors by activating the tumor necrosis factor-α (TNF-α)/nuclear factor kappa B (NF-κB) axis and transform growth factor β (TGF-β). DFO promoted migration in the ROS-dependent NF-κB and TGF-β signaling pathway by enhancing mitochondrial iron accumulation in TNBC (Liu et al., 2016). Although preclinical studies have confirmed that iron chelating agents inhibit tumor growth and metastasis by certain signaling pathways. However, in clinical studies, iron chelating agents only show moderate therapeutic benefits, and may promote anti-tumor drug resistance due to ferroptosis. It is urgent to improve the bioavailability of iron chelating agents and combination therapy.

Some Chinese medicine and natural compounds have been shown to induce ferroptosis in breast cancer by targeting iron overload. Shuganning induces ferroptosis *via* Heme oxygenase-1(HO-1) to increase intracellular iron accumulation and suppresses tumor growth in TNBC (Du et al., 2021). Artemisinin is activated by Fe^2+^ to generate free radicals. The iron supplements can improve the anticancer efficacy of artemisinin. Artemisinin can induce ferroptosis by promoting ferritin phagocytosis and thus increasing intracellular iron levels (Chen et al., 2020). A completed phase I clinical trial (NCT00764036) showed that for patients with metastatic breast cancer, oral artemisinin of up to 200 mg per day was deemed safe and well tolerated (von Hagens et al., 2017).

### 5.2 Ferritinophagy activators

Ferritinophagy serves as a link between ferroptosis and autophagy (Latunde-Dada, 2017). Ferritinophagy transports ferritin to the lysosome for degradation. The release of free iron leads to increased iron, and the Fenton reaction occurs to generate oxidative stress, which is essential for ferroptosis. Autophagic degradation reduces the expression of the ferritin storage protein. Decreased ferritin expression promotes the onset of ferroptosis. The BRD4 inhibitor (+)-JQ1 (JQ1) confer ferroptosis by autophagy in MDA-MB-231 cells. JQ1 was shown to induce ferritin phagocytosis or decreased the expression of GPX4, SLC7A11, and SLC3A2 (Sui et al., 2019). Dihydroartemisinin (DAT) combined with RSL3 was used to treat breast cancer by targeting ferritin. DAT induces lysosomal degradation of ferritin and impact the iron regulatory protein (IRP)/iron response element (IRE) controlled iron homeostasis to promote the level of cellular free iron (Chen et al., 2020).

### 5.3 System Xc–inhibitors

System Xc–, a glutamate-cystine antiporter, is consists of the light chain subunit SLC7A11 and the heavy chain subunit SLC3A2. SLC3A2 anchors SLC7A11 to the plasma membrane and maintains the stability of SLC7A11 (Liu M. R. et al., 2021). Several compounds-such as erastin, sulfasalazine, and sorafenib—have been utilized as inhibitors of SLC7A11. The erastin is a classic ferroptosis agonist that inhibits the function of System Xc- by irreversibly binding to SLC7A11 and inactivating SLC7A11, resulting in the depletion of GPX4 and GSH. The erastin-induced ferroptosis by increasing cellular lipid ROS in TNBC (Wang H. et al., 2022). Fascin directly interacts with System Xc- and decreases its stability *via* ubiquitination. Fascin enhances anti-tumor effect to erastin-induced ferroptosis (Chen C. et al., 2022). Sulfasalazine (SAS) can active ROS production to induce the ferroptosis in breast cancer.

Metformin increases Fe^2+^ and lipid ROS, reduces the stability of SLC7A11 by inhibiting the ultrafiltration process of SLC7A11, and metformin combined with SAS can synergistically activate ferroptosis and inhibit the growth of breast cancer cells (Yang et al., 2021). The pH regulator carbonic anhydrase IX (CAIX) induced by hypoxia prevents ferroptosis in cancer cells, and co-targeting of CAIX/XII and xCT induce ferroptosis in TNBC. The novel synthetic lethal interaction will help researchers develop new strategies for solid tumor therapy (Chafe et al., 2021). p53 and BRCA1 Associated Protein 1 (BAP1) inhibit carcinogenesis in part by antagonizing SLC7A11 and induce ferroptosis (Zhang Y. et al., 2019). Eupaformosanin was shown to induce ferroptosis by ubiquitination of the mutant p53 in TNBC (Wei et al., 2022).

### 5.4 The agents to suppress the GPX4 pathway

GPX4 is an essential enzyme for the elimination of lipid hydrogen peroxide. GPX4 reduces the production of ROS radicals in membrane lipids and protects normal breast cells from ROS DNA damage to inhibit ferroptosis. GPX4 inhibitors include RSL3, the RSL3 analogs DPI7, DPI12, DPI13, DPI17, DPI18 and DPI19, ML210, JKE1647, altretamine, FIN56, FINO2, dihydroartemisinin, and Withaferin A. RSL3 inactivates GPX4, leading to ferroptosis by ROS accumulation (Stockwell, 2022). Erastin also suppresses the expression of GPX4 in breast cancer, especially in TNBC. Vitamin C induces TNBC cells death by suppressing GPX4. Furthermore, sequential treatment with VC and quercetin (Q) significantly increased ROS. A study suggested that vitamin C and Q can be developed as an adjuvant agents for cancer patients by Nrf2 (Mostafavi-Pour et al., 2017). Statins, the mevalonate pathway inhibitors, are identified as ferroptosis agents that target HMGCR. Statins suppress CoQ10 production to induce ferroptosis and decrease GPX4 (Gobel et al., 2019). Lycium barbarum polysaccharide (LBP), a traditional Chinese medicine, promotes ferroptosis in breast cancer by modulating the xCT/GPX4 pathway (Du et al., 2022). Ketamine inhibits GPX4 levels and inhibits histone H3 lysine 27 acetylation (H3K27ac) and enrichment of RNA polymerase II (RNA pol II) by attenuating KAT5 on the GPX4 promoter in breast cancer cells (Li H. et al., 2021). Glycyrrhetinic acid (GA) induces lipid peroxidation to trigger ferroptosis in TNBC cells by reducing the activity of GSH and GPX4 (Wen et al., 2021). DMOCPTL can induce EGR1 mediated apoptosis and ferroptosis and in TNBC by GPX4 ubiquitination (Ding et al., 2021). Researchers screened drug combinations using a synthetic lethal mechanism (simultaneous inhibition of two or more non-lethal genes leading to cell death) and found that low doses of targeted drugs targeting BET and cytoplasmic proteins *in vitro* could reduce the levels of GPX4 and trigger ferroptosis in TNBC (Verma et al., 2020).

### 5.5 Ferroptosis-based nanotherapy in breast cancer

Nanomedicine is a favorable solution for the development of ferroptosis because of its better efficacy and improved targeting ability, low systemic toxicity, and high safety (Zafar et al., 2021). The mechanism of emerging nanomedical therapy is mainly including: 1) triggering Fenton reaction; 2) inhibiting the expression of GPX4; 3) exogenous regulation of lipid peroxidation. The diversified multifunctional nanomaterials can produce H_2_O_2_, eliminate GSH, and provide Fenton reaction catalyst. In addition, some iron-containing nanomaterials can also induce ferroptosis. Azo-CA4 was loaded into nanocarriers and is converted by near-infrared light to ultraviolet light. After irradiation, Azo-CA4-loaded nanocarriers reduced the viability of TNBC through ferroptosis (Zhu et al., 2020). Sonodynamically amplified ferroptotic red blood cells (SAFEs), a state-of-the-art red blood cell was engineered for simultaneous activation of ferroptosis and oxygen-enriched sonodynamic therapy (SDT). SAFE contains internalized RGD peptide and erythrocyte membrane mixed with camouflaged hemoglobin nanocomplex, perfluorocarbon, sonosensitizer verteporfin, and FINS (dihomo-gamma-linolenic acid, DGLA). SAFE can not only provide abundant oxygen to the hypoxia-related therapeutic resistance of the defect by ultrasound stimulation, but also activate ferroptosis by the combination of ROS production and lipid peroxidation, which selectively accumulates and induces desirable anticancer effects in the tumor (Zhou A. et al., 2022). Heparinase-driven continuous release nanoparticles (HSPE) can enhance the killing power of tumor cells *via* DOX-induced ferroptosis, regulating the tumor microenvironment, and effectively inhibiting breast cancer metastasis (Zhang J. et al., 2021). The lapatinib and pseudolaric acid B (PAB) were delivered by ferritin-containing nanoparticles, which showed an enhanced tumor suppression ability by increasing ROS (Wu X. et al., 2022).

A tumor-targeted MOF nanosheet system with multienzyme-like catalytic activity can inhibit the antiferroptotic mechanism mediated by GPX4 and FSP1 in tumor cells, thus enhancing ferroptotic damage. After iRGD promoted tumor cells to uptake Au/Cu-TCPP(Fe)@RSL3-PEG-iRGD nanosheets, besides the ability of RSL3 to activate GPX4, the glucose oxidase (Gox) activity of gold nanoparticles could easily comsume intracellular glucose and inhibit the production of NADPH, resulting in reduced expression of GSH and GPX4 (Li K. et al., 2022). Rosuvastatin (RSV) was loaded into silk fibroin (SF) nanoparticles (NPs) (Cu-SF(RSV), to disrupt redox stabilization regulated by the CoQ10/FSP1 axis by interfering with the mevalonate pathway to improve ferroptosis sensitivity in TNBC (Yang J. et al., 2022). In one study, the drug simvastatin (SIM) was loaded into zwitterionic polymer-coated magnetic nanoparticles (Fe_3_O_4_@PCBMA). PCBMA promoted the accumulation of Fe3O4@PCBMA in tumor lesions, while SIM inhibited the expression of HMGCR, thereby downregulating mevalonate pathway and GPX4 expression, and inducing ferroptosis (Yao et al., 2021). Cinnamaldehyde dimers (CDCs) induce lipid materials to form dimers, which enable them to deplete GSH and deliver therapeutics to enhance ferroptosis and immunotherapeutic effects against breast cancer (Zhou Z. et al., 2022). The nanoparticles consisting of ferritin and a pH-sensitive molecular switch (FPBC@SN) disintegrate in the acidic cytoplasm and release sorafenib and an indoleamine2,3-dioxygenase (IDO) inhibitor NLG919. NLG919 reduces tryptophan metabolism by inhibiting IDO, thus improving tumor immunity and antitumor efficacy. Sorafenib upregulates nuclear receptor coactivator 4 (NCOA4), which produces lipid peroxide (LPO) through ferritin autophagy. Down-regulation of GPX4 and LPO clearance elicit the ferroptosis in tumor cells (Zuo et al., 2021).

The combination of immunotherapy and ferroptosis death nanoparticles can also play an ideal therapeutic effect on breast cancer. A bionic Fe_3_O_4_ magnetosome (PaM/Ti NC), TGF-β Inhibitor (Ti) is carried in the membrane and PD-1 antibody (Pa) is anchored on the outer surface of the nanoparticles. The level of intracellular H_2_O_2_ can be increased under immunogenic microenvironment, thus the ferroptosis can be triggered by Fenton reaction. The ideal combined anti-tumor effect is achieved through the mutual promotion of ferroptosis and immunomodulation (Zhang F. et al., 2019).

## 6 Combination therapy for breast cancer

Combining ferroptosis inducers with immunotherapy and radiation therapy may be a suitable strategy to enhance ferroptosis in breast cancer and make tumors sensitive to immunotherapy and radiation therapy, especially for those with high expression of antiferroptosis genes or low ferroptosis-promoting genes (Sun et al., 2021). Furthermore, ferroptosis has been confirmed to induce synergetic crosstalk between immunotherapy and RT (Table 2).

**TABLE 2:** The combination therapy with FINS in breast cancer.

Treatment	Agents	Target	Mechanism	Subtype	References
Target-therapy	Lapatinib and Siramesine	iron	Increasing the intracellular iron level	TNBC	Ma et al. (2016)
Radio-therapy	Holo-Lf& RT	ROS	increase the production of lipid ROS	TNBC	Zhang et al. (2021c)
	Laser irradiation and Gallic acid	GPX4, Lipid peroxide-tion	Improving anticancer effects of gallic acid through inhibiting GPX4	TNBC	Khorsandi et al. (2020)
	γ-radiation and Erastin	System Xc-	Enhancing GSH synthesis to regulate ROS	TNBC	Cobler et al. (2018)
Immuno-therapy	Dichloroacetate and Albiziabioside A	GPX4	Inhibiting GPX4 and reprogramming the polarization of M2-TAMs	Luminal A	Wei et al. (2019)
	PDT and FINS		Inducing lymphocytes infiltration in the tumor and stimulates the secretion of IFN-γ	Mice BC	Xu et al. (2020)
Chemo-therapy	Deferasirox and Cisplatin, Doxorubicin	Iron overload	Increasing the intracellular iron level	BC	Tury et al. (2018)
	Saponin formosanin and Cisplatin	GPX4	Lipid ROS accumulation and GPX4 deletion	BC	Zafar et al. (2021)
Nanomedcine and Biomaterial	Au/Cu-TCPP(Fe)@RSL3-PEG-iRGD	GSH	Depleting intracellular glucose and inhibit the production of NADPH	TNBC	Li et al. (2022b)
	Cu-SF(RSV)	CoQ10/FSP1	Interfering with the mevalonate pathway	TNBC	Yang et al. (2022b)
	Azo-CA4	iron	Reducing Fe3+ to Fe 2+	TNBC	Zhu et al. (2020)
	SAFEs	ROS	ActiveDGLA -mediated lipid peroxidation,	BC	Zhou et al. (2022a)
	Fe3O4@PCBMA	HMGCR	Downregulating mevalonate pathway and GPX4 expression,	TNBC	Yao et al. (2021)
	HSPE	ROS	Enhancing intercellular ROS	Mice BC	Zhang et al. (2021b)
	CDCs	GSH	Depletion of GSH and enhance dendritic cells maturation	Mice BC	Zhou et al. (2022b)
	FPBC@SN	LPO GPX4	which produces lipid peroxide (LPO) through ferritin autophagy	BC	Zuo et al. (2021)

### 6.1 Target therapy with ferroptosis inducers in breast cancer

Lapatinib is a potent double tyrosine kinase inhibitor of the epidermal growth factor receptor (EGFR) and HER-2 which inhibits the proliferation of breast cancer cells with overexpression of ErbB2 and EGFR (Guarneri et al., 2021). Siramesine is a lysosomotropic agent that releases cathepsin B from lysosomes, leading to increased ROS production and cell death (Petersen et al., 2013). The combination of lapatinib and siramesine could induce ferroptosis in TNBC by boosting intracellular Fe2+ (Ma et al., 2016).

### 6.2 Radiotherapy in combination with ferroptosis inducers

RT is a common cancer treatment that uses the ionizing radiation (IR) to eradicate cancer cells (Delaney et al., 2005). Binding of FINS targeting GPX4 or SLC7A11 to RT can sensitize breast cancer cell lines or xenografts to radiation by enhancing ferroptosis sensitivity. Zhang et al. found that iron-saturated lactoferrin (holo-lactoferrin, Holo-Lf) significantly decreased GPX4 expression in MDA-MB-231 cells and cell viability, and combination therapy with erastin could further inhibit cell viability. The combination of Holo-Lf with RT can significantly increase the production of lipid ROS in MDA-MB-231 cells, indicating that RT can promote cell ferroptosis and improve radiosensitivity (Zhang et al., 2021c). Pre-red laser irradiation combined with gallic acid could enhance anticancer effects by decreasing GPX4 activity (Khorsandi et al., 2020). γ-radiation combined with erastin improves GSH synthesis and controls ROS generation, the therapeutic effectors of radiation therapy (Cobler et al., 2018). Knocking down ESR1 expression may enhance the efficacy of radiation-induced ferroptosis *via* the NEDD4L/CD71 pathway in breast cancer (Liu et al., 2022). Although the combination of RT and FINS appears to be safe in preclinical studies, some researches have suggested that ferroptosis may also be associated with RT-induced damage of normal tissue (Li et al., 2019). Therefore, it is urgent to clarify whether this combination therapy has negligible toxicity to normal tissues.

### 6.3 Immunotherapy together with ferroptosis inducers

Dichloroacetate (DCA) combined with albiziabioside A (AlbA) can inhibit GPX4 and induce ferroptosis to inhibit tumor progression (Wei et al., 2019). DCA can also improve glycolytic lactate immunosuppression and promote the effect of immunotherapy (Ohashi et al., 2013). AlbA has the characteristic of selective antitumor effect without affecting normal cells (Wei et al., 2014). The synergistic effect of the AlbA-DCA conjugate which can affect the polarization of M2-TAM to reprogram the tumor immune microenvironment and ferroptosis will enhance therapeutic efficacy.

Photodynamic therapy (PDT) gather lymphocyte infiltration in tumor lesions and stimulates IFN-γ secretion (Xu et al., 2020). Hemoglobin (Hb) can be used as an oxygen provider in PDT due to its excellent oxygen-carrying properties and can also be used as an iron supplement in the treatment of ferroptosis due to the iron component (Wang et al., 2015). Synergistically induced ferroptosis in cancer cells could be a potential strategy for treating breast cancer (Xu et al., 2020).

### 6.4 Chemotherapy combined with ferroptosis inducers

The development of various FINS and inhibitors has accelerated research on the ferroptosis and chemosensitivity. ACSL4 in tumor lesions can be used as a predictive factor of complete pathological remission and progression-free survival of neoadjuvant chemotherapy in breast cancer. Importantly, the imbalance between ACSL4/GPX4 can indicate the level of ferroptosis after undergoing neoadjuvant chemotherapy, effectively distinguishing patients with different complete pathological response rates and disease-free survival. With the increase in ACSL4 and the decrease in GPX4, the pathological complete remission rate of patients has gradually increased (Sha et al., 2021).

The iron chelator deferasirox can synergistically enhance the efficacy of doxorubicin and cisplatin in TNBC without increasing side effects (Tury et al., 2018). Saponin formosanin C (FC) is a potent FIN that showed the strongest cytostatic effect among all anticancer drugs, including cisplatin, using the MDA-MB-231 cell line as a model (Chen H. C. et al., 2022). The excessive intake of dietary iron leads to the production of oxidative stress and DNA damage. A collaborative group clinical trial revealed that the use of antioxidant supplements during chemotherapy, such as iron and vitamin B12, increased intracellular iron to serve as a propellant and could increase recurrence and mortality in breast cancer. This finding should be considered when discussing the use of dietary supplements during chemotherapy with breast cancer patients (Ambrosone et al., 2020).

## 7 Future perspectives

As a hotspot in tumor research, ferroptosis plays a crucial role in a variety of tumor cells. New therapeutic targets have the potential to stimulate the development of strategies for the treatment of tumors. Research on ferroptosis in breast cancer focuses on the cellular level *in vitro*, which provides a relevant theoretical basis for clinical application.

Despite the rapid progress of ferroptosis research in breast cancer, some challenges remain to be elucidated, including the identification of the best candidate drugs and targeting of ferroptosis in clinical trials. FINS are used in combination with other therapeutic approaches, such as immunotherapy or RT, which may induce mixed RCD to inhibit tumor growth. How to select the subtype of breast cancer and subgroups of patients who are sensitive to ferroptosis for targeted treatment should also be elucidated.

Genomics provides a platform for identifying the gene mutations that drive tumor progression and constitute therapeutic targets. Therefore, the integration of genetic information may help distinguish tumors that respond (or do not respond) to ferroptosis. If the expression of pro-apoptotic genes is low but that of ferroptosis-promoting genes is high, patients may derive more benefit from ferroptosis drugs. In addition, depending on the vulnerability of specific breast cancer tumors, different FINS can be used to target endogenous or exogenous pathways.

## 8 Conclusion

Ferroptosis is considered as a double-edged sword, and the potential toxic side effects of inducers or inhibitors should be fully elucidate to ensure the activation of the Fenton response and avoid off-target toxicity to normal organ and tissue. In the future, the rational design of safe and effective ferroptosis-based antitumor drugs should be mutifaceted. There are still significant challenges in basic research and clinical translation based on ferroptosis. However, we believe that with the advancement of basic research on ferroptosis, it will be possible to translate FINS or enhancers into clinical practice of breast cancer therapy.

## Glossary

AA: Arachidonic acid
ACSL: acyl CoA synthase long chain family member
ADA: Adrenaline
ADHFE1: Alcohol Dehydrogenase Iron Containing 1
AlbA: Albiziabioside A
ALOX5: Arachidonate 5-Lipoxygenase
AKR1C1: Aldo-keto reductase family 1member C1
BAP1: BRCA1 associated protein 1
BC: breast cancer
BRCA1: BRCA1 associated protein 1
BRD4: Bromodomain containing 4
CAIX: carbonic anhydrase IX
CDCs: Cinnamaldehyde dimers
COQ10: ubiquinone
CSC: cancer stem cell
CISD2: CDGSH Iron Sulfur Domain 2
DAT: Dihydroartemisinin
DCA: Dichloroacetate
DDR2: discoidin domain receptor tyrosine kinase 2
DFO: Deferoxamine
DFX: desferoxazole
DGLA: dihomo-gamma-linolenic acid
DHFR: dihydrofolate reductase
DHODH: Dihydroorotate dehydrogenase (Quinone)
DKK1: Dickkopf WNT Signaling Pathway Inhibitor 1
DMT1: divalent metal transporter 1
DMT1: divalent metal transporter 1,
EGFR: epidermal growth factor receptor
EGR1: early growth response 1
EMT: Epithelial-to-mesenchymal transition
ESR1: estrogen receptor 1
FC: formosanin C
Fins: ferroptosis inducers
FPN: ferroportin,
FRG: Ferroptosis-related gene
FSP1: Ferroptosis suppressor protein
FTH1: ferritin heavy chain 1
FTL: ferritin light chain
FGFR4: Fibroblast Growth Factor Receptor 4
GA: Glycyrrhetinic acid
GCH1: Guanosine triphosphate cyclohydrolase 1
GCL: γ-glutamylcysteine ligase
GFRA1: GDNF Family Receptor Alpha 1
GGT1: gamma-glutamyltransferase 1
Gox: glucose oxidase
GPX4: glutathione peroxidase 4
GS: Glutathione synthetase
GSH: Glutathione
GSK-3 β: Glycogen synthase kinase-3 β
H3K27ac: H3 lysine 27 acetylation
Hb: Hemoglobin
HLF: hepatic leukemia factor
HMGB1: high mobility group box 1
HMGCR: 3-Hydroxy-3-methylglutaryl coenzyme A reductase
HO-1: Heme oxygenase-1
Holo-Lf: holo-lactoferrin
H2Bub1: H2B monoubiquitination
IDO: indoleamine2,3-dioxygenase
IFN-γ: interferon-γ
IR: ionizing radiation
IRE: iron response element
IRP: iron regulatory proteins
LBP: Lycium barbarum polysaccharide, Lipopolysaccharide Binding Protein
LOX: lipoxygenase
LPCAT3: lysophosphatidylcholine acyltransferase 3
LPO: lipid peroxide
MUFA: Mono-unsaturated Fatty Acid
NCOA4: nuclear receptor coactivator 4
NF-κB: nuclear factor-κB
Nrf2: nuclear factor erythroid
NQO1: NADH Dehydrogenase Quinone 1
OTUB1: OTU Deubiquitinase, Ubiquitin Aldehyde Binding 1
PDT: Photodynamic therapy
PE: phosphatidylethanolamine
PGM5P3-AS1: PGM5P3 antisense RNA 1
PLOOH: phospholipid hydroperoxide
PLS: phospholipids
POR: P450 oxidoreductase
PUFA-PL: polyunsaturated fatty acid phospholipids
PKCβII: Protein kinase C-βII
RCD: regulated cell death
RHOT1: Ras homolog family member T1
RNA pol II: RNA polymerase II
ROS: reactive oxygen species
RSV: Rosuvastatin
RT: radiotherapy
SAFE: Sonodynamically amplified ferroptotic red blood cells
SAS: Sulfasalazine
SDT: sonodynamic therapy
SF: silk fibroin
SIM: simvastatin
SLC3A2: Solute carrier family 3 member 2
SLC7A11: Solute carrier family 7 member 11
SOD: superoxide dismutase
SRF: sorafenib
STAT3: signal transducer and activator of transcription 3
TAMs: Tumor-associated macrophages
TF: transferrin,
TFR1: Transferrin Receptor 1
TFR1: transferrin receptor 1,
TGF-β2: transforming growth factor-beta 2
TME: tumor microenvironment
TNBC: triple-negative breast cancer
TNF-α: tumor necrosis factor-α
VC: Vitamin C
27HC: 27-hydroxycholesterol
USP7: ubiquitin-specific protease 7
